# Bayesian Estimation of the Probability of Virologic Failure on Cabotegravir and Rilpivirine Long-Acting in Real Life

**DOI:** 10.7759/cureus.76829

**Published:** 2025-01-02

**Authors:** Sebastian Noe, Anna Ivanova, Farhad Schabaz, Ariane E von Krosigk, Carmen Wiese, Eva Wolf, Celia Jonson-Oldenbuettel

**Affiliations:** 1 HIV Research and Clinical Care, Medizinisches Versorgungszentrum München am Goetheplatz, Munich, DEU; 2 I-BioStat, Hasselt University, Hasselt, BEL; 3 Clinical Research, Medizinisches Versorgungszentrum München am Goetheplatz, Munich, DEU

**Keywords:** dual antiretroviral therapy, hiv-1, injection-site reaction, long-acting injectable, real-world data, virologic failure

## Abstract

Background

Virologic failure (VF) is still a major concern in the use of cabotegravir (CAB) and rilpivirine (RPV) long-acting (LA) for many healthcare professionals (HCP). While many results from clinical trials have been published, there is suspicion that they might underestimate the risk under less-controlled real-life conditions.

This study aimed to estimate the probability of VF (primary objective) as well as discontinuation for any reason (secondary objective) among people with HIV (PWH) on CAB and RPV LA every two months (Q2M) in real life using Bayesian methodology.

Methods

Bayesian estimation of VF based on prior knowledge about VF and discontinuation on CAB and RPV Q2M from randomized controlled trials (ATLAS-2M, SOLAR) and real-world data from CAB and RPV LA from a large single-center cohort.

Results

Among 175 PWH, two (1.1%) met the criteria of VF through week 48 (W48), resulting in an estimated risk of VF at W48 of 1.2% [0.6%; 1.9%] using Bayesian estimation. In one of the PWH, two-class resistance was observed at the time of VF, most likely being therapy emerging.

The probability of discontinuation for any reason by W48 was 21.4%, leading to a Bayesian risk estimate of 8.9% [7.3%; 10.5%]. The main reasons for discontinuation were injection-site reactions (n=10).

Conclusions

Risk of VF on CAB and RPV LA under real-life conditions seems to be comparable to results in clinical trials. This finding can be reassuring for both PWH and HCPs considering CAB and RPV LA as an alternative to oral antiretroviral treatment, particularly when considering the risk factors for VF that have been identified from the cases of VF in the clinical trials for patient selection.

At the same time, rates of discontinuation may be considerably higher. However, this does not seem to be an indicator of a worse safety profile outside clinical trials, but probably could be the result of making CAB and RPV LA available to a wider population of PWH.

## Introduction

Cabotegravir (CAB) and rilpivirine (RPV) long-acting (LA) have become the first complete non-oral antiretroviral therapy (ART) regimen, approved for routine use in people with HIV (PWH) without resistance toward non-nucleoside/nucleotide reverse transcriptase (NNRTI) or integrase inhibitors (INI) or a history of virologic failure (VF) under an INI- and/or NNRTI-containing regimen.

While for many, CAB and RPV LA is a promising regimen for PWH struggling with daily intake of ART for various reasons, it is also faced with considerable skepticism, particularly from healthcare providers. There is concern about the ability of PWH to hold appointments in a time frame of ±7 days around the due day of the injection [1], in order to avoid sub-therapeutic drug concentrations that could favor the development of resistance. Adding to this concern, the development of VF with and without therapy-emerging resistance has been observed in the clinical trial program [2-5]. Another specific concern around injected drugs is the occurrence of injection-site reactions (ISRs) that might contribute to high rates of discontinuation.

This study aimed to estimate the probability of VF in particular, as well as discontinuation for any reason in general based on data from two-monthly (Q2M) CAB and RPV LA in a real-world cohort, incorporating prior findings from the Q2M arm of the ATLAS-2M, as well as the SOLAR trials [3,6], using Bayesian methodology.

## Materials and methods

Study design

A retrospective, single-center, longitudinal, observational study in a large outpatient HIV research and clinical care center (Medizinisches Versorgungszentrum München am Goetheplatz) in Munich, Germany. The use of anonymized clinical routine data from a single center did not require the Ethics Committee’s approval.

Inclusion and exclusion criteria

The inclusion criterion was having received at least one dose of CAB and RPV LA after December 2020; people being on CAB and RPV for less than 48 weeks and who had not experienced VF or discontinuation for any reason, people participating in clinical trials, or who never received at least one dose of CAB and RPV LA were excluded from the analysis. 

Objectives and outcome measures 

The primary outcome measure was VF (two consecutive plasma HIV1-RNA concentrations >200 copies/mL and/or discontinuation due to virologic inefficacy). Primary and secondary objectives were the estimation of the probabilities of VF and discontinuation for any reason at week 48 (W48) after CAB and RPV LA initiation, respectively.

Statistics

Using Bayesian methodology, the likelihoods of VF and discontinuation in the study were assumed to follow binomial distributions, with the risk set containing all PWH who had a follow-up of at least 48 weeks after initiation or discontinued before W48 (for reasons of comparability to the results from the snapshot analyses on the intention-to-treat-exposed (ITT-E), which were used for the construction of the prior). Conjugate beta priors were derived from the combined W48 results of the Q2M arm in ALTAS-2M [6], as well as the SOLAR [3] trial with the ITT-E population as the risk set for VF and discontinuation at W48, respectively. The beta-binomial posterior distributions were approximated using Markov-Chain Monte Carlo methods with 5,000 iterations and a burn-in period of 1,000 for each of 10 chains. Mixing was investigated visually using trace plots while the stationarity of the chains was tested using Gelman-Rubin’s diagnostic for multiple chains. More details on the methodology can be found in the Appendix. Sensitivity analyses were performed using priors from ATLAS-2M (pessimistic approach) as well as SOLAR (optimistic approach) only (e.g. without combining the findings). 

All analyses were performed using R 4.2.0 (R Foundation for Statistical Computing, Vienna, Austria). For all analyses, parameter estimates together with 95% highest posterior density credibility intervals were calculated. Sensitivity analyses were performed by using both, an optimistic as well as a pessimistic prior based on the published data.

## Results

**Figure 1:**
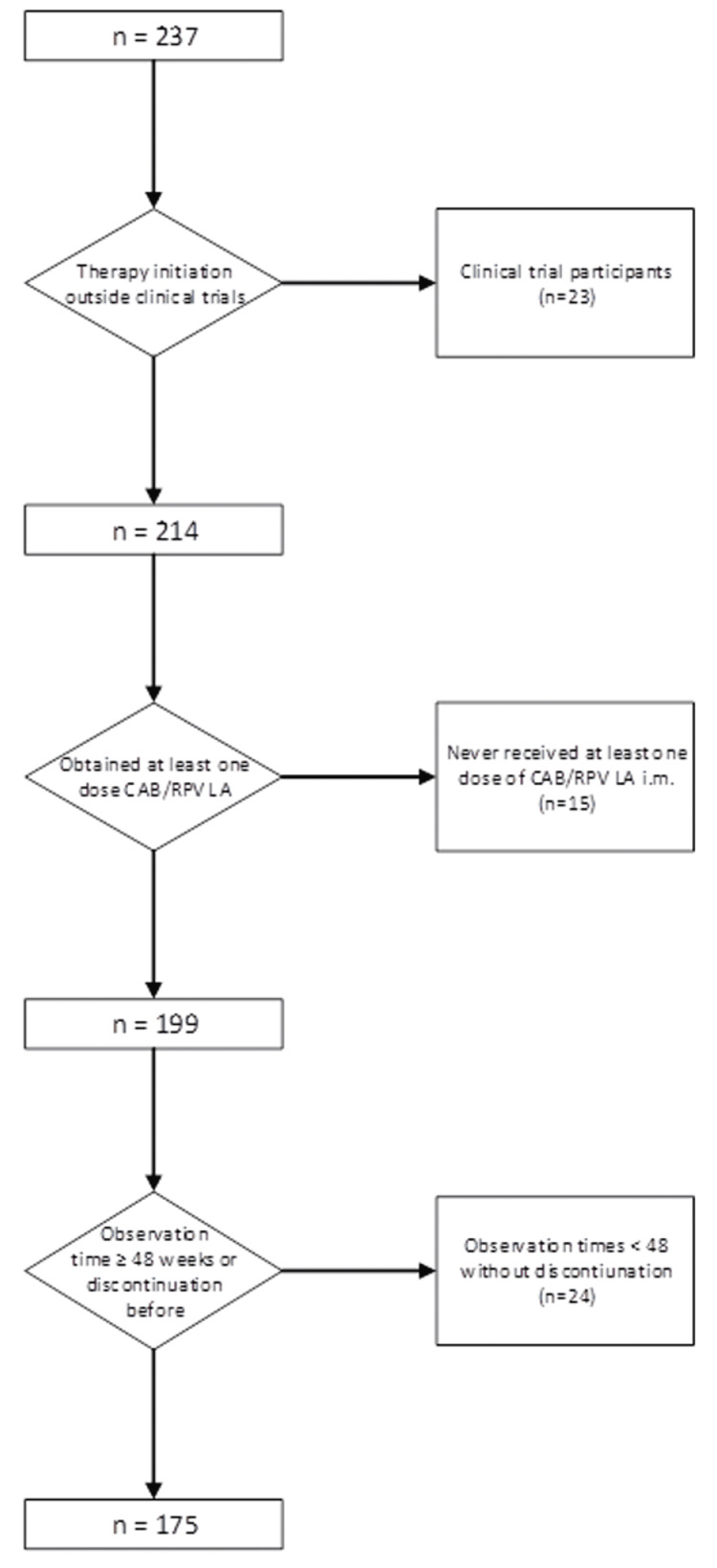
Sequential application of inclusion and exclusion criteria for patient selection. CAB: cabotegravir; RPV: rilpivirine; LA: long-acting.

Overall, 237 PWH were prescribed CAB and RPV between December 2020 and October 2023; 175 remained after applying inclusion and exclusion criteria (Figure 1) and were used as the risk set for the primary analysis (Table 1). Most PWH initiating CAB and RPV had HIV-1 RNA <50 copies/mL; the highest HIV-1 RNA at first injection was found to be 82 copies/mL.

**Table 1:** Characteristics of the risk set for the primary objective at the time of first injection. CD4: cluster of differentiation 4; INI: integrase inhibitor; MSM: man who has sex with men; NNRTI: non-nucleoside/nucleotide reverse transcriptase inhibitor.

			n
Age (years), median (IQR)	45	(36; 53)	175
Sex (male), n (%)	149	(85.1%)	175
Ethnicity (Caucasian), n (%)	132	(75.4%)	175
Route of transmission (MSM), n (%)	91	(79.1%)	115
Prior exposure to NNRTIs, n (%)	83	(47.4%)	175
Prior exposure to INIs, n (%)	144	(82.3%)	175
HIV1-RNA <50 copies/mL at first injection, n (%)	148	(98.7%)	150
CD4 cells at first injection (cells/µL), n (%)	746	(587; 975)	150
CD4 nadir (cells/µL), median (IQR)	323	(168; 427)	88
History of AIDS (yes), n (%)	9	(7.3%)	124
HIV-1 subtype (B), n (%)	78	(73.3%)	101

Based on the results from the Q2M arm of ATLAS-2M and SOLAR trials, a β (12, 973) distribution was derived as the prior (details can be found in the Appendix). In our study sample, two PWH (1.1%) met the criteria of VF through W48 (occurring at months 5 and 9, respectively), resulting in a binomial (175, 0.011) likelihood, leading to a posterior probability of VL at W48 of 1.2% [0.6%; 1.9%].

For the secondary objective, i.e. discontinuation for any reason through W48, three PWH were excluded, who had planned to have a one-time CAB and RPV LA application only. Based on the reported discontinuation, a β (71, 910) distribution was derived as the prior (details can be found in the Appendix). In our study, 37 (21.4%) discontinued by W48, resulting in a binomial (172, 0.214) likelihood, leading to a posterior probability of discontinuation at W48 of 8.9% [7.3%; 10.5%].

Reasons for discontinuation are displayed in Table 2. Among the local side effects, there was one abscess that led to discontinuation, while all other ISRs were pain with or without swelling and reddening and in general not severe (n=10). Among the systemic side effects, there was one immediate reaction to the injection that was considered either allergic or due to inadvertent intravascular injection. Other systemic side effects were headaches (n=1), worsening of a depressive episode (n=2), fatigue (n=1), impaired liver function tests (n=1), as well as pyrexia (n=1). Two discontinuations were due to suspected adverse events without further information. Preemptive discontinuation included discontinuation for previously unnoticed proviral resistance with a possible effect on RPV and/or CAB (n=6), problems in adherence (n=1), and long-time unavailability of injections (n=2). The recorded death was not ART-related and occurred in a state of advanced malignant disease.

**Table 2:** Reasons for discontinuation of cabotegravir and rilpivirine.

	n (%)
Local side effects	11 (29.7)
Systemic side effects	9 (24.3)
Preemptive discontinuation	9 (24.3)
Patient preference	4 (10.8)
Virologic failure	2 (5.4)
Potential drug-drug interaction with comedication	1 (2.7)
Death	1 (2.7)

For a Bayesian sensitivity analysis, we varied the prior assumptions as follows: For a conservative approach to estimating the rate of VF, the prior was only based on the Q2M arm of the ATLAS-2M trial, which led to a posterior probability of VF of 1.6% [0.7%; 2.5%] while basing the analysis on the prior information from SOLAR only, it resulted in 1.0% [0.3%, 1.7%].

## Discussion

Our findings support the notion of VF being a rare event under CAB and RPV LA Q2M even outside clinical trials with two out of 175 PWH (1.1%) in the study sample fulfilling the criteria at W48. As estimating an incidence of rare events is generally imprecise, Bayesian methodology lends itself to estimating a probability of VF, taking previous information from clinical trials and our real-world findings into account. Combining the pooled prior knowledge with our data in a Bayesian manner, an updated probability of VF of 1.2% [0.6; 1.9] was estimated. This implies that VF does not occur more often in clinical routine than in clinical trials. During the 2024 Conference on Retroviruses and Opportunistic Infections and in medical journals, several data were published, reporting rates of VF under CAB/RPV LA in real-life settings as well as a clinical trial in Africa of 0.4-4.0% [7-10]. These findings might be highly relevant in decision-making for CAB and RPV LA, as a frequent concern particularly among healthcare professionals might be the fear of a (much) higher rate of VF in the real world and lead to the fact that fewer people are given the possibility to profit from LA as an alternative to oral ART. 

For discontinuation for any reason, rates of 6.9% and 9.5% were reported in ATLAS-2M and SOLAR through W48, respectively, while we observed a discontinuation rate as high as 21.4%. The discrepancy is, however, probably not surprising and can very likely be related to a stricter selection of PWH that enter clinical trials. Offering the possibility to experience CAB and RPV LA in clinical routine more widely to PWH who demonstrate interest, probably despite some doubts, will therefore very likely lead to a higher rate of discontinuation. We do not see the increased rate of discontinuation as a sign of an adverse safety profile in clinical routine; the degree of ISRs that contributed most to the discontinuations was not more severe than in the center’s long-standing experience in clinical trials but led more often to discontinuation.

Our study has several limitations. Despite having a considerable number of PWH on CAB and RPV LA included in this analysis, the sample size is still small compared to the ATLAS-2M and SOLAR trials. Therefore, the impact of the real-world findings is dominated by the prior, which in particular for the question on discontinuation might be relevant, as prior and study data lead to markedly different results and are therefore in conflict to some extent. Furthermore, being a single-center experience, our data may not reflect the heterogeneity of real-world conditions throughout different centers. In particular, being a center with early implementation and a very patient-centered approach to medical care with a liberal attitude toward innovations, a higher rate of discontinuation might have been expected with a higher number of PWH being given the opportunity to experience CAB and RPV LA even in cases where people might have been unsure. Also, it must be kept in mind that many people were taken off CAB and RPV preemptively due to previously unknown findings (including pre-existing resistance-associated mutations in proviral resistance tests); one might (rightfully) argue that these people might not have been started on CAB and RPV LA in the first place and that these PWH contributed relevantly to the excess probability of discontinuation. Further research might use bigger cohorts with a wider variety of PWH but also clinical settings to obtain more robust estimates of both VF as well as discontinuation for any reason.

## Conclusions

Our data demonstrate that the rate of VF in the real world does not seem to exceed the expectations derived from the results of clinical trials, despite a less-controlled setting and a more diverse population given the opportunity to experience CAB and RPV LA. While there might therefore be higher rates of discontinuation, they are not driven by high rates of VF or severe adverse events. The most frequent reasons for discontinuation were ISRs and patient decisions, which could be seen as the result of balanced, informed decisions of PWH, in whom the benefit of LA therapies does not outweigh their downsides; this remains, however, speculative. On the other hand, several preemptive discontinuations (for example, due to medical details that only got available after the switch, such as resistance tests) might have led to a higher rate of discontinuation in real life than in clinical trials; in the setting of a clinical trial, these patients would very likely not have entered the trial in the first place. A more detailed comparison between the real-world data and the clinical trial data could be used to investigate whether the "threshold for discontinuation" is lower in real life than in the clinical trials or if the safety profile of CAB and RPV LA is adverse in clinical routine. While the analyses are dominated by the prior probabilities due to the high number of PWH included in the underlying trials, it seems recommendable to collect further real-world data from a broader range of PWH from multicenter cohorts to obtain a more stable estimate for both efficacy and safety of CAB and RPV in clinical routine.

## Figures and Tables

**Figure 2:**
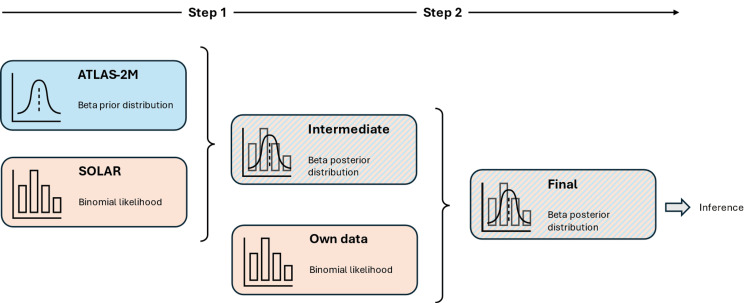
Flow chart on the step-by-step application of Bayesian methodology to obtain a final estimate for the probabilities of virologic failure and discontinuation based on the results of two clinical trials (ATLAS-2M and SOLAR) and the data obtained from the study sample of the current study.

**Figure 3:**
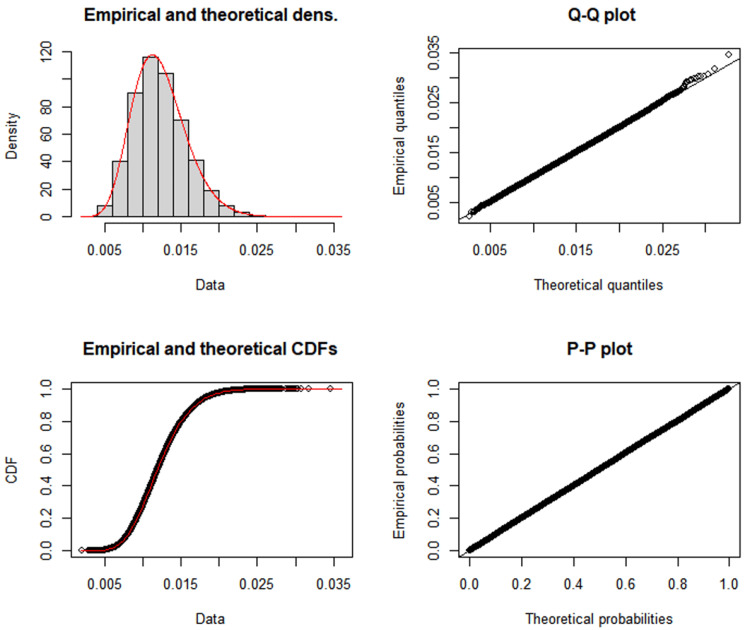
Illustration of the fit of the beta distribution of the probability of virologic failure with parameters α=12 and β= 973 to the posterior distribution of the combined findings from ATLAS-2M and SOLAR. CDF: cumulative distribution function; P-P: probability-probability; Q-Q: quantile-quantile.

**Figure 4:**
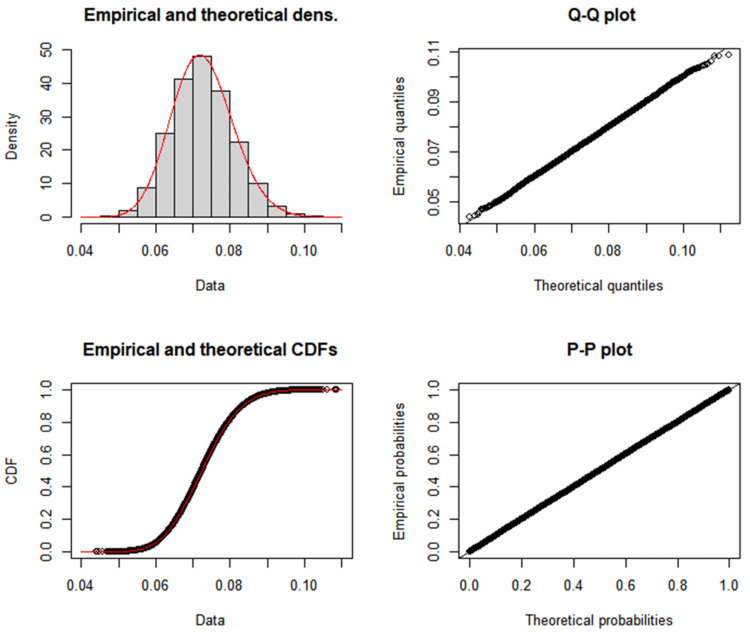
Illustration of the fit of the beta distribution of the probability of discontinuation with parameters α=71 and β=910 to the posterior distribution of the combined findings from ATLAS-2M and SOLAR. CDF: cumulative distribution function; P-P: probabilitiy-probability; Q-Q: quantile-quantile.

## Appendices

General procedure of the development of the prior distribution used in the study

In the first step, the prior probability distributions from the previously published results of ATLAS-2M (Q2M arm) were combined with the likelihood of the SOLAR trial (both at W48) for each of the endpoints separately. Parameters for the beta distribution were calculated as x + 1 and N-x+1, with N being the risk set and x being the number of events. The resulting beta distribution was then used as the conjugate prior for the binomial likelihood of the data from the study sample collected in this study. The combination of the two resulted in a final beta distribution that was used for inference (Figure 2).

Development of the prior distribution for the primary objective (virologic failure)

In the two-monthly dosing arm of the ATLAS-2M trial, eight PWH with VF were reported at week 48 out of the ITT-E population (n=522), translating into a β(9, 515) prior. The likelihood was derived from the 48-week result of the SOLAR trial (mITT-E), resulting in a B(454, 0.0066) distribution. A beta-distribution was fitted to the resulting posterior, using maximum likelihood, resulting in the parameter estimates α=12 and β= 973. This posterior distribution was used as the updated prior for the data analysis from the observations in the study.

Development of the prior distribution for the secondary objective (discontinuation)

In the two-monthly dosing arm of the ATLAS-2M trial, 36 PWH discontinued CAB/RPV LA through week 48 out of the ITT-E population (n=522), translating into a β(35, 489) prior. The likelihood was derived from the 48-week result of the SOLAR trial (mITT-E), resulting in a B(454, 0.0793) distribution. A beta-distribution was fitted to the resulting posterior, using maximum likelihood, resulting in parameter estimates α=71 and β=910. This posterior distribution was used as the updated prior for the data analysis from the observations in the study.
